# Fibromyalgia Syndrome and Cognitive Decline: The Role of Body Mass Index and Clinical Symptoms

**DOI:** 10.3390/jcm11123404

**Published:** 2022-06-14

**Authors:** Cristina Muñoz Ladrón de Guevara, Gustavo A. Reyes del Paso, María José Fernández Serrano, Casandra I. Montoro

**Affiliations:** 1 Department of Psychology, University of Jaén, 23071 Jaén, Spain; greyes@ujaen.es; 2 Department of Methodology for Behavioral Science, University of Granada, 18071 Granada, Spain; mjfser@ugr.es

**Keywords:** body mass index, clinical pain, cognitive decline, fibromyalgia

## Abstract

The high prevalence of obesity and overweight in fibromyalgia (FM) may be an important factor in the well-known cognitive deficits seen in the disorder. This study analyzed the influence of body mass index (BMI) and primary clinical symptoms of FM (pain, fatigue, insomnia, anxiety, and depression) on attention, memory, and processing speed in FM. Fifty-two FM patients and thirty-two healthy participants completed cognitive tasks assessing selective, sustained, and divided attention; visuospatial and verbal memory; and information processing speed. Furthermore, they were evaluated in terms of the main clinical symptoms of the disorder. FM patients showed a marked reduction of cognitive performance in terms of selective, sustained, and divided attention; visuospatial memory; and processing speed, but no group differences were observed in verbal memory. BMI negatively affects sustained and selective attention, verbal memory, and processing speed and is the main predictor of performance in these basic cognitive domains. Our findings confirm the presence of cognitive deficits with respect to attention and visual memory, as well as slower processing speed, in FM. Moreover, the results support a role of BMI in the observed cognitive deficits. Interventions increasing physical activity and promoting cognitive stimulation could be useful for strengthening cognitive function in FM patients.

## 1. Introduction

Fibromyalgia (FM) is defined as a chronic pain syndrome of unknown etiology characterized by diffuse, widespread, and non-inflammatory musculoskeletal pain, which is accompanied by symptoms such as morning stiffness, fatigue, mood disorders, sleep disturbances, and cognitive impairment; it predominantly affects middle-aged adult women, that is, women over 50 years of age [1,2,3]. One of the most common complaints reported by FM patients is cognitive alterations [4,5,6,7]. Specifically, these cognitive complaints have been suggested to affect 50–80% of FM patients and include memory loss that extends to the capacity to recall names, conversations, words and quotations, problems expressing thoughts, difficulty in adequately remembering directions and scheduled activities, and a kind of “fog” that prevents sufferers from perceiving daily events clearly [8,9,10]. These cognitive problems, together with the experience of pain, limit the daily life activities of FM patients and cause a great deal of discomfort [11,12]. In fact, patients report that they are among the most deleterious symptoms of the disease, due to their negative impact on functional capacity, working life, and quality of life [13,14,15].

Despite patients’ complaints, cognitive dysfunction in FM has received relatively less clinical and empirical support/attention compared with clinical pain symptoms; however, more studies have appeared in recent years, with current evidence indicating the presence of cognitive deficits in several neuropsychological domains [13,16]. Although short- and long-term implicit and working memory deficits, as well as slowness to complete complex cognitive tasks (i.e., executive control and emotional recognition tasks) have been confirmed [9,13,16,17,18,19,20,21,22], the results remain controversial. While most studies point to general deficits in information processing speed; selective and sustained attention; and visuospatial, verbal, and semantic memory in FM patients compared to healthy participants [5,13,18,23,24,25,26,27,28,29], others observed no substantial cognitive deficits in association with FM [27,30,31,32,33,34]. 

In addition, there is no consensus on the factors influencing the cognitive impairments seen in FM, although the intensity of clinical pain has been proposed as one such factor. Several studies have shown an inverse association between clinical pain levels and cognitive performance in FM patients (e.g., [16,19,28,31,35,36,37,38,39,40]). However, it is important to note that not all studies demonstrated this association [10,23,41,42].

Other factors proposed to explain the presence of cognitive impairments in FM include medication intake, mood and emotional alterations (i.e., depression and anxiety), and fatigue and sleep problems. However, while some studies have reported a significant relationship between depression and/or anxiety, and cognitive deficits in FM [25,38,42,43,44], others suggested that cognitive deficits are independent of comorbid depression and/or anxiety disorders [5,6,16,28,29,39]. The situation is similar for fatigue and/or sleep disorders, i.e., some studies have supported the notion that these factors influence cognitive performance [27,28,45], while others have not [19,29,39,40,46,47]. 

Body mass index (BMI) may also influence cognitive performance, especially when meeting the threshold for obesity (i.e., BMI > 25 kg/m^2^; [48,49,50,51]. Indeed, this has been demonstrated in the general population [52,53,54]. However, although overweight and obesity are highly prevalent in FM [55,56], to the best of our knowledge, only two studies have attempted to elucidate the relationship between BMI and cognitive impairment in FM. The first one, performed by Soriano-Maldonado et al. [57], revealed strong associations between aerobic fitness, attention, working memory, delayed recall, and verbal learning in FM, while no associations were observed between parameters used to assess overweight (BMI, body fat percentage, fat mass index, and waist circumference) and cognitive ability. Contrarily, Muñoz Ladrón de Guevara et al. [16] found a negative influence of BMI on performance in a cognitive test measuring components of executive function (updating, inhibitory control, switching, decision-making, self-regulation, and planning) in FM patients. 

Given that findings regarding the negative association between BMI and cognitive function in FM are limited to executive function components, and taking into account the equivocal results regarding the deficits in basic cognitive processes (attention, memory, and processing speed) seen in FM, the aims of the present study were to: (1) test for deficits in basic cognitive processes in FM, in the domains of attention (selective, sustained, and divided attention), memory (verbal and visuospatial), and information processing speed; and (2) explore the association between BMI and these basic cognitive processes. The role of clinical variables such as pain, anxiety, depression, fatigue, sleep problems (insomnia), and medication use on the cognitive performance of FM patients will be also explored.

## 2. Materials and Methods

### 2.1. Participants

The study was part of a larger project assessing cognition and emotional processing in FM [16,21,58]. In total, 52 patients diagnosed with FM and 32 healthy participants took part in the study. Given the higher prevalence of FM in women compared to men and with the aim of avoiding possible gender-related confounding, only women were included in the study. Patients were recruited through the Fibromyalgia Associations of Jaén and Úbeda (Spain). All of the patients met the American College of Rheumatology criteria for the diagnosis of FM [3]. Healthy participants were recruited through local advertisements. Exclusion criteria for all participants included metabolic abnormalities, neurological disorders (e.g., traumatic head injury), and severe somatic (e.g., cancer) or psychiatric (e.g., drug dependency, psychosis) diseases. The sociodemographic and clinical data are shown in Table 1. Patients did not differ from healthy participants in terms age or years of education but showed a trend toward a higher BMI.

### 2.2. Clinical Assessments

The clinical history and sociodemographic data of the patients were obtained via a semi-structured interview. The Structured Clinical Interview for Axis I Disorders of the Diagnostic and Statistical Manual of Mental Disorders (SCID [59]) was used to check for psychiatric disorders, especially anxiety and depression. Clinical pain was quantified using the Spanish version of the McGill Pain Questionnaire (MPQ [60]). The following four scales from the MPQ were applied: sensory pain (score range: 0–84), affective pain (score range: 0–22), evaluative pain (score range: 0–4), and miscellaneous pain (score range: 0–30). The internal consistency (Cronbach’s α) of the Spanish version of the MPQ ranges from 0.66 to 0.80 [61]. Depressive symptoms were assessed using the Spanish version of the Beck Depression Inventory (BDI [62]; score range: 0–63; Cronbach’s α = 0.95). Levels of anxiety were assessed using the State-Trait Anxiety Inventory (STAI [63]; score range: 0–60 for both scales; Cronbach´s α = 0.93 for state anxiety and 0.87 for trait anxiety). Fatigue was assessed using the Spanish version of the Fatigue Severity Scale (FSS [64]; score range: 9–63; Cronbach´s α = 0.88). Insomnia was measured using the Oviedo Sleep Quality Questionnaire (COS [65]; score range: 9–45; Cronbach´s α = 0.77).

### 2.3. Cognitive Assessment

The ***d2 Attention test*** (d2 [66]) was used for the measurement of sustained and selective attention. The d2 consists of a cancellation task in which participants must discriminate between (target stimuli) and cancel (non-target stimuli) visually similar stimuli. It consists of 658 items divided into 14 rows, each consisting of 47 characters. Specifically, it has 16 different types of characters, i.e., the letters “d” and “p”, each with one, two, three or four small quotation marks. The target stimulus is the letter “d” with two quotation marks (““) that appear together (above or below the letter “d”) or separately. The letter “d” accompanied by one, three, or four consecutive quotation marks and the letter “p” (regardless of the number of quotation marks) act as distractor stimuli. The duration of the entire test ranges from 8 to 10 min. The participant has 20 s to respond to each of the test rows. The d2 variables indexing performance are as follows: the number of stimuli or items attempted in the 14 rows (TR_d2); total test effectiveness (d2_TOT), calculated as the total number of items attempted in the 14 rows (TR) minus the total number of omissions and commissions; and the concentration index (d2_CON), calculated as the number of items correctly marked minus the number of errors of commission.

The ***Trail Making Test*** (TMT [67]) was used for measuring divided attention and information processing speed. The TMT is a subtest of the Delis–Kaplan Executive Function System (D-KEFS [67]) battery. It consists of a set of five conditions composed of visual stimuli (letters and numbers), preceded by a test trial. The conditions are as follows: Condition 1 (visual scan; participants must identify the three numbers that appear on the answer sheet), Condition 2 (number sequencing; participants must line up numbers 1 to 16 in ascending order while ignoring the letters), Condition 3 (letter sequencing; participants must line up letters A to P in alphabetical order while ignoring the numbers), Condition 4 (switching between numbers and letters; participants must connect numbers and letters in alphanumeric order (numbers in ascending order and letters in alphabetical order, i.e., 1-A, 2-B...until reaching 16-P)) and Condition 5 (motor speed; participants must draw a dotted line). The stimuli that comprise each of these five conditions appear in a larger area than in the original version of the TMT, such that there is more interference [67]. For all five conditions, the examiner instructs the participants to respond as quickly and precisely as possible. Performance on Condition 4 was taken as a measure of divided attention [68] and performance on Conditions 2 and 3 as a measure of information processing speed [42,69]. 

The ***Rey–Osterrieth Complex Figure Test*** (ROCF [70]) was used to measure visuospatial memory. The ROCF consists of three conditions: copying, immediate recall, and delayed recall. In the first condition, the ROCF figure is presented, and the participants are asked to copy it. Immediately thereafter, in the second condition, participants have to remember and draw the figure without any visual guidance, and after a 30 min delay (third condition), the figure must be drawn once again. The scores vary according to the scoring system used (maximum = 36 points) but are typically based on evaluations related to location, accuracy, and organization (18 items). Each ROCF condition takes 5 min to complete, and the total test time is approximately 40 min. Copy accuracy (first condition: copy execution) and copy reproduction from memory (delayed; third condition: memory execution) were taken as performance measures.

The ***Five-digit test*** (5DT [71]) was used for measuring information processing speed. This test consists of four conditions presented in order of difficulty (the least difficult first). In each condition, a series of 50 stimuli framed by small rectangles (each rectangle contains one to five digits or asterisks) are presented. In Condition 1 (reading), the participant is asked to read the digits, while in Condition 2 (counting) the asterisks have to be counted. In Condition 3 (interference), the digits must be counted while ignoring the numerical values (note that the number of digits in the boxes does not correspond to their numerical values). Finally, in Condition 4 (alternation), the criteria are identical to those in Conditions 1 and 2 (reading, counting), but participants must switch from the primary to the secondary criterion (i.e., from counting to reading) according to a visual cue. Test performance was indexed by the spent taken (information processing speed) to complete each condition [72]; shorter times indicate better performance.

The ***Test de Aprendizaje Verbal Español-Complutense*** (TAVEC [73]) was used to measure verbal memory. In the first condition of the TAVEC, the evaluator reads aloud a list of 16 words (List A; also called the shopping list) five times, and participants must immediately recall as many words as possible (immediate free memory). Following this, the evaluator reads aloud a new list (List B; interference list), which the participant must also reproduce. After a 20 min of break, a third list of 44 words is read out. This list includes all words from List A and several from List B, as well as some distractor words not included in either previous list. The participant must decide whether each word belongs to List A (recognition task). The performance indices are the total number of words recalled during the five trials (RI_AT), total number of words recalled in the short-term recall trial (RL_CP), and total number of words recalled in the long-term recall trial (RL_LP).

### 2.4. Procedure

All participants were evaluated individually in two sessions approximately 2 h in duration separated by 1 week. In the first session, a clinical psychologist recorded the patients’ clinical history, sociodemographic data (including weight and height), and medication intake and determined whether they met any of exclusion criteria. Subsequently, the SCID was conducted, during which the questionnaires previously described in the clinical assessment section were completed. In addition, in order to detect possible simulated memory impairment, participants completed the 15-item Rey Memory Test [70]; none of the participants met the criterion for impairment (score < 6). The cognitive tests were performed during the second session. The tests were presented in a fixed order, alternating between verbal and nonverbal tasks. There was a 5 min break after the completion of each cognitive task. The study was approved by the Human Research Ethics Committee of the University of Jaén and all participants gave written informed consent.

### 2.5. Statistical Analysis

Group differences in cognitive performance were tested for by multivariate analysis of variance (MANOVA), including BMI as a covariate and then by univariate ANOVA models (also including BMI as a covariate). The effects of medication use and comorbid depression and anxiety disorders were subjected to stratified analyses in the FM group only, using MANOVA models comparing patients using and not using analgesics, anxiolytics, opioids, and antidepressants, as well as patients with and without depressive and anxiety disorders (with BMI as a covariate). The effect sizes are indicated by adjusted eta squared ($\eta_{p}^{2}$) values.

Associations between clinical variables and cognitive performance were only analyzed in the FM group. Firstly, to reduce the number of correlations performed (and thus limit type I error), we performed a multiple correlation analysis (the correlation coefficient (R) indicates the existence of an association, but not its direction (positive or negative), between the predictor variables (anxiety [STAI], depression [BDI], fatigue [FSS], insomnia [COS], and the four clinical pain variables [MPQ]) and each cognitive domain (selective attention and sustained attention [d2_TR, d2_CON, d2_TOT of the d2 Attention test], divided attention [Condition 4 of the TMT], visuospatial memory [copying and memory conditions of the ROFC], verbal memory [RI_AT, RL_CP, RL_LP of the TAVEC], and information processing speed [Conditions 2 and 3 of the TMT, and Conditions 1–4 of the 5DT]). Secondly, multiple regression analyses using the stepwise method were conducted, with BMI and the clinical variables as predictors and the cognitive parameters as dependent variables. The adjusted $R^{2}$ was used to evaluate the changes in predictions associated with each new block.

## 3. Results

### 3.1. Demographic and Clinical Variables

FM patients reported higher rates of depression and anxiety, and of self-reported clinical pain, depression, anxiety, fatigue and insomnia, than healthy participants. Additionally, FM patients used more opioid and non-opioid analgesics, anxiolytics, and antidepressants than healthy participants (Table 1).

#### Group Comparisons

The MANOVA revealed a significant main effect of group on general cognitive performance (F[15, 67] = 2.20, *p* = 0.015, $\eta_{p}^{2}$ = 0.33), but no main effect of BMI (F[15, 67] = 1.69, *p* = 0.075, $\eta_{p}^{2}$ = 0.27). 

Table 2 displays the means and standard deviations of the cognitive parameters. Univariate analysis showed significant group differences in all performance variables, except information processing speed (Condition 2 of both the TMT and 5DT) and all TAVEC verbal memory conditions; the FM patients showed poorer performance than the healthy participants. A significant main effect of BMI was also observed on selective and sustained attention (d2_TR, d2_CON, d2_TOT), information processing speed (Condition 2 of the TMT and Conditions 2–4 of the 5DT), and verbal memory (RI_AT, RL_CP, and RL_LP conditions of the TAVEC). Higher BMI was associated with poorer cognitive performance for these conditions.

The MANOVAs performed to compare cognitive performance between FM patients with and without depression or anxiety disorders (SCID) did not reveal a significant main effect of the presence of depression (F[15, 35] = 0.69, *p* = 0.775, $\eta_{p}^{2}$ = 0.23), but there was a trend toward better performance by patients with anxiety disorders (F(15, 35) = 1.96, *p* = 0.051,$\;\eta_{p}^{2}$ = 0.46). However, in univariate analysis, the effect of the presence of anxiety disorders was non-significant for all measured cognitive variables. Moreover, in multivariate analysis, there was no main effect of medication use (F[15, 35] = 1.00, *p* = 0.476, $\eta_{p}^{2}$ = 0.30 for anxiolytics; F[15, 35] = 0.57, *p* = 0.875, $\eta_{p}^{2}$ = 0.20 for analgesics; F[15, 35] = 1.17, *p* = 0.339, $\eta_{p}^{2}$ = 0.33 for antidepressants; and F[15, 35] = 0.52, *p* = 0.914, $\eta_{p}^{2}$ = 0.18 for opioids). 

### 3.2. Associations between Clinical Variables and Cognitive Performance

#### 3.2.1. Exploratory Multiple Correlation Analysis

Exploratory multiple correlation analysis revealed an association between BMI and selective and sustained attention (d2 Attention test [d2_TR, d2_CON and d2_TOT]; *R* = 0.46, *p* = 0.009) and information processing speed (TMT Conditions 2 and 3 and 5DT Conditions 1–4; *R* = 0.52, *p* = 0.021). In addition, the different pain indices (MPQ) were associated with visuospatial memory (ROCF copying and memory conditions; *R* = 0.48, *p* = 0.001 for sensorial pain; *R* = 0.46, *p* = 0.003 for affective pain; and *R* = 0.49, *p* = 0.001 for miscellaneous pain). No other associations were observed in the multiple correlation analysis.

#### 3.2.2. Regression Analysis

Table 3 shows the results of the multiple regression analysis performed to determine the ability of BMI and the clinical variables to predict cognitive performance. Regarding selective and sustained attention, BMI negatively predicted performance in the d2_TR, d2_CON and d2_TOT conditions. Regarding information processing speed, BMI (in the first model) and sensorial pain (in the second model) were positively associated with the time taken to perform TMT Condition 2; moreover, the time taken to perform Conditions 3 and 4 of the 5DT was positively predicted by BMI. With respect to visuospatial memory, memory execution (ROCF) was negatively predicted by sensorial pain (MPQ). Regarding verbal memory, the RI_AT TAVEC condition was positively predicted by state anxiety (STAI), and the RI_CP TAVEC condition was negatively predicted by BMI. Finally, state anxiety was positively associated (in the first model) with sensorial pain and negatively related (in the second model) to the RI_LP TAVEC condition.

## 4. Discussion

This study explored the cognitive domains of attention, memory, and information processing speed in FM patients using a neuropsychological test battery that comprehensively assesses selective, sustained, and divided attention, visuospatial and verbal memory, and cognitive processing speed. In addition, we explored the influence of BMI and clinical variables such as pain, anxiety, depression, fatigue, insomnia, and medication use on the cognitive performance of these patients.

Compared to healthy participants, FM patients showed lower performance in the domains of selective and sustained attention, divided attention, processing speed, and visuospatial memory. These results support the notion of markedly impaired attention, visuospatial memory, and information processing speed in FM and are in accordance with previous studies [5,18,23,28,29,74,75]. However, in opposition to our preceding findings [58] no group differences were observed in verbal memory (as measured by the TAVEC). Although at first glance the lack of differences in verbal memory between FM and healthy participants may seem striking, the literature on this matter is in fact inconsistent. Indeed, and in accordance with the present findings, Castel et al. [30,76] did not find differences in verbal memory (as measured by the TAVEC) between FM patients and controls. These discrepant results may be explained in part by differences in the dependent variables (i.e., TAVEC conditions) selected to index verbal memory performance. In the present study, we selected three measures of immediate free recall, as well as measures of short- and long-term recall, while in our previous study [58], two measures of immediate free recall, errors of omission, false-positive responses, and an index of discrimination were used. It is also important to mention that, in the present study, the effect of BMI was controlled for in the group comparisons, and that BMI significantly affected verbal memory performance (see below a further discussion of this issue). In conclusion, not all studies have demonstrated differences in verbal memory between FM and healthy participants (e.g., [27,30,32,76] for negative results). More research is needed to shed light on the verbal memory performance of FM patients.

Significant effects of BMI on selective and sustained attention, information processing speed, and verbal memory were also found in this study; higher BMI was associated with lower performance in these cognitive domains. By contrast, BMI did not significantly affect divided attention or visuospatial memory. Correlation and regression analysis confirmed that higher BMI was associated with lower performance in FM. Specifically, higher BMI predicted slower processing speed, less sustained and selective attention, and poorer verbal memory. However, BMI was not associated with variables indexing divided attention and visuospatial memory.

The findings regarding BMI are of special importance, as obesity and overweight are often seen in FM [55]. A higher BMI has been related to more severe FM and to the occurrence of musculoskeletal pain in the general population [55,77]. The complex relationships between obesity and pain may be mediated by mechanical overload and multiple proinflammatory and neurohormonal mechanisms [78]. In agreement with a recent review by D’Onghia et al. [55] revealing a high prevalence of obesity in European FM patients, the mean BMI in our FM sample was 28.29 kg/m^2^ (class 1 obesity; [51]). Moreover, higher rates of pain complaints and chronic pain have been reported among individuals with obesity [79].

Obesity has been cited a risk factor for poor cognitive performance in the general population [80], especially in the domains of attention/vigilance, visual and verbal memory, information processing speed, and executive function [53,54,81,82,83,84]. Our results confirm the role of BMI in cognitive deficits in FM, including not only complex cognitive functions (i.e., executive function) as reported in a previous study (see Muñoz Ladrón de Guevara et al. [16]), but also more basic cognitive processes such as sustained and selective attention, verbal memory, and processing speed. The precise mechanisms linking cognitive performance and obesity have not yet been identified, although altered brain structure, blood–brain barrier and leptin regulation, poorer cerebrovascular function and blood flow cerebral perfusion, arterial hypertension, oxidative stress, and inflammation have also been implicated [80,85,86,87,88,89,90,91,92].

Against this background, the mechanisms underlying the relationship between obesity and pain might explain the detrimental effect of BMI on cognitive performance in FM. Several pronociceptive and antinociceptive pathways have suggested to have an important role in the relation between obesity and pain sensitivity, particularly the presence of inflammatory markers such as interleukin-6 (IL-6) and C-reactive protein (CRP) [93]. Interestingly, Okifuji et al. [56] found that, in women with FM, CRP was positively associated with BMI. Additionally, higher CRP levels have been associated with poor memory, attention, and processing speed in clinical samples [94].

Similarly, elevated levels of leptin (a hormone synthesized in adipose tissue and produced in excess in obese subjects) have been associated with body pain in healthy postmenopausal women with a BMI > 25 kg/m^2^ and in FM patients whose average weight was 81.17 kg/m^2^ [95]. Peripheral leptin penetrates the cerebrospinal fluid and central nervous system and interacts with the hypothalamus and hippocampus [96,97], which are thought to be altered in FM [98,99]. The involvement of the hippocampus in memory processes is well known, and leptin has been implicated in memory impairment in obese populations [85]. From a behavioral perspective, physical inactivity and a sedentary lifestyle may be contributing factors. Physical inactivity is frequent in FM, which promotes obesity and impaired cognitive performance [100,101]. Increased physical activity, specifically an increase in aerobic capacity, is one of the most effective methods for treating FM [102]. Several studies have shown an association between aerobic exercise and improvements in attention, processing speed, executive function, and memory in the general population [103,104].

Regarding the effect of clinical factors on the cognitive performance of FM patients, our results showed a negative association between the different pain components (sensorial, affective, and miscellaneous) and visuospatial memory. Regression analysis confirmed the association for sensorial pain. These findings corroborate the well-known interfering effect of pain on cognitive function in FM [16,19,28,29,36,39,47].

Although our FM patients with and without a diagnosis of depression or anxiety did not differ in cognitive performance, the regression analysis showed that state anxiety was a positive predictor of some TAVEC verbal memory variables. This association of cognitive improvement with state anxiety is striking, as other studies either suggested a negative influence of anxiety on working memory, attention, and general cognitive function [42,43,105], or no effect of anxiety [16,19,28,29,39]. This result is difficult to explain, but it is possible that higher state anxiety promotes greater arousal, where increased activation can lead to improved performance [106,107]. Finally, our non-significant results for fatigue, insomnia, and medication use (for all four types of drugs) are in line with previous studies suggesting a minor role of these factors in the cognitive impairment seen in FM patients [40,47,108].

A limitation of the present study was the relatively small size of the patient sample, as well as the smaller number of participants in the healthy group relative to the FM patient group. This might have limited the statistical power of the group comparisons. Nevertheless, our sample size was comparable to or larger than those in most other studies in this field [25,26,27,40,108]. Additionally, another limitation of this study pertains to the lack of information about the possible influence of medication on the assessed variables; this could have been explored by comparing patients grouped according to the use of particular medications or combinations thereof. The sample size was insufficient to form such patient subgroups, although previous studies did not suggest substantial effects of medication on cognitive performance in FM [40]. Nonetheless, it is important to highlight that previous literature has reported some side effects on cognition and weight associated to the medication use. For instance, an increase of greater cognitive deterioration [109] and significant weight gain [110,111] have been associated to anticonvulsants use in adults. Furthermore, factors such as fatigue, mental effort, and distraction during performance of the tests were not controlled for, similar to most other studies. These factors can impact the cognitive performance of FM patients [23,112]. Likewise, given the inverse association revealed in this study between the experience of clinical pain and cognitive performance in FM, and the lack of research evaluating differences between FM severity subgroups on these factors, it should be considered as a future line of research. Finally, although the relevance of BMI to the cognitive performance of our FM patients is clear, possible mediating mechanisms, such as physical exercise and general level of fitness, were not measured and should be considered in future research.

## 5. Conclusions

In conclusion, our findings confirm the presence of deficits in cognitive basic processes, such as selective, sustained, and divided attention; visuospatial memory; and information processing speed (but not verbal memory), in FM patients. Moreover, BMI negatively affected the cognitive performance of our patients. In light of the high prevalence of overweight and obesity in FM [55], our findings suggest the need for interventions to reduce the body weight of these patients. In particular, tailored physical exercise is strongly recommended for FM. Interventions improving physical capacity, disability, and fatigue could increase cognitive performance directly, as well as indirectly via the associated reduction in body weight. Finally, given the empirical support for the presence of cognitive deficits in FM, neuropsychological rehabilitation programs for these patients are recommended.

## Figures and Tables

**Table 1:** Means (M) and standard deviations (SD) of the clinical and sociodemographic data of fibromyalgia (FM) patients and healthy controls. Results of group comparisons (t or χ2) are also displayed.

	FM (N = 52) M ± SD or n (%)	Healthy Controls (N = 32) M ± SD or n (%)	t [82]/χ2	*p*
Age (y)	51.25 ± 8.67	52.94 ± 6.59	−0.95	0.35
Years of education	9.27 ± 3.52	10.59 ± 3.64	−1.65	0.10
BMI	28.29 ± 4.49	26.49 ± 4.36	1.80	0.075
Depression (SCID), n, %	22 (42.30)	2 (6.25)	12.62	<0.0001
Anxiety Disorders * (SCID), n, %	25 (48.08)	7 (21.88)	5.77	0.016
Antidepressant use, n, % Anxiolytic use, n, %	27 (51.92) 35 (67.31)	2 (6.26) 8 (25.00)	18.28 14.19	<0.0001 <0.0001
Non-opioid analgesic use, n, % Opiate use, n, %	45 (86.54) 23 (44.23)	8 (25.00) 0 (0.00)	32.22 16.49	<0.0001 <0.0001
State anxiety (STAI)	30.92 ± 11.92	17.19 ± 9.60	5.51	<0.0001
Trait anxiety (STAI)	35.29 ± 9.34	17.56 ± 10.24	8.14	<0.0001
Depression (BDI)	21.90 ± 12.56	4.47 ± 5.67	7.39	<0.0001
Fatigue (FSS)	50.56 ± 12.35	19.88 ± 11.14	11.92	<0.0001
Insomnia (COS)	29.73 ± 7.43	17.09 ± 7.52	7.91	<0.0001
Sensory pain (MPQ) Affective pain (MPQ)	35.59 ± 18.39 5.92 ± 4.50	12.38 ± 3.85 0.71 ± 0.72	8.52 8.10	<0.0001 <0.0001
Evaluative pain (MPQ) Miscellaneous (MPQ)	3.27 ± 1.03 9.25 ± 5.90	2.29 ± 1.38 4.14 ± 2.56	3.33 5.16	<0.01 <0.0001

* Note: Anxiety disorders include panic disorder, generalized anxiety disorder, phobias and adjustment disorder; n = number of participants. Non-opioid analgesic use includes the following analgesic drugs: non-steroidal anti-inflammatory drugs, 29 patients; paracetamol, 34 patients; metamizole, 7 patients; anticonvulsants, 10 patients; tramadol, 20 patients; and codeine, 4 patients.

**Table 2:** Means (M) and standard deviations (SD) of cognitive performance parameters for the FM patients and healthy controls: F, *p* and $\eta_{p}^{2}$ values indicating the main effects of group and body mass index (BMI) are also presented.

Test	FM N = 52 M ± SD	Healthy Controls N = 32 M ± SD	F[1, 81] for Group	*p* for Group	$\eta_{p}^{2}\;\mathrm{for}\;\mathrm{Group}$	F[1, 81] for BMI	*p* for BMI	$\eta_{p}^{2}\;\mathrm{for}\;\mathrm{BMI}$
d2_TR	347.56 ± 77.45	407.63 ± 91.95	7.46	<0.01	0.08	7.85	<0.01	0.09
d2_CON	111.08 ± 47.87	150.22 ± 36.69	12.55	<0.01	0.13	4.99	0.028	0.06
d2_TOT	313.23 ± 81.17	385.47 ± 86.77	11.42	<0.01	0.12	8.90	<0.01	0.10
TMT Condition 2	53.38 ± 26.99	45.25 ± 17.76	0.97	0.328	0.01	8.38	<0.01	0.10
TMT Condition 3	75.62 ± 43.02	51.75 ± 25.28	6.39	0.013	0.07	2.10	0.151	0.03
TMT Condition 4	159.60 ± 67.49	122.03 ± 53.55	5.89	0.017	0.07	0.98	0.326	0.01
ROCF copying condition	29.30 ± 6.40	33.83 ± 3.01	12.42	<0.01	0.13	0.53	0.470	0.01
ROCF memory condition	15.77 ± 6.16	20.36 ± 6.44	8.60	<0.01	0.10	2.13	0.148	0.03
5DT Condition 1	23.90 ± 6.94	19.72 ± 3.05	9.05	<0.01	0.10	0.52	0.473	0.01
5DT Condition 2	27.52 ± 9.26	23.72 ± 4.14	3.19	0.078	0.04	4.19	0.044	0.05
5DT Condition 3	46.67 ± 18.10	36.41 ± 6.10	6.80	0.011	0.08	8.29	<0.01	0.09
5DT Condition 4	62.19 ± 22.69	49.91 ± 13.03	4.84	0.031	0.06	15.39	<0.0001	0.16
TAVEC RI_AT	50.31 ± 9.65	53.44 ± 10.78	0.90	0.346	0.01	5.03	0.028	0.06
TAVEC RL_CP	10.88 ± 2.83	10.97 ± 3.08	0.012	0.726	0.00	6.07	0.016	0.07
TAVEC RL_LP	11.12 ± 2.76	11.66 ± 2.75	0.15	0.695	0.00	6.33	0.014	0.07

Note: d2 = d2 Attention test; D-KEFS = Delis–Kaplan Executive Function Test Battery Trail Making Test; ROCF = Rey–Osterrieth Complex Figure Test; 5FDT = Five-Digit Test; TAVEC = Test de Aprendizaje Verbal Español-Complutense.

**Table 3:** Results of multiple regression analysis performed to determine the ability of clinical factors and BMI to predict cognitive performance in FM patients.

Cognitive Test	Model	Predictor	β	r^2^	t	*p*
d2_TR	Model 1	BMI	−0.42	0.17	−3.24	0.002
d2_CON	Model 1	BMI	−0.28	0.08	−2.08	0.043
d2_TOT	Model 1	BMI	−0.42	0.18	−3.28	0.002
TMT Condition 2	Model 1	BMI	0.38	0.15	2.94	0.005
	Model 2	BMI	0.35	0.11	2.85	0.006
		Sensorial Pain (MPQ)	0.33		2.67	0.010
5DT Condition 3	Model 1	BMI	0.32	0.10	2.35	0.023
5DT Condition 4	Model 1	BMI	0.49	0.24	3.92	<0.0001
ROCF memory condition	Model 1	Sensorial Pain (MPQ)	−0.46	0.21	−3.65	<0.01
TAVEC RI_AT	Model 1	State anxiety (STAI)	0.32	0.10	2.38	0.021
TAVEC RL_CP	Model 1	BMI	−0.28	0.08	−2.02	0.048
TAVEC RL_LP TAVEC	Model 1	State anxiety (STAI)	0.30	0.09	2.18	0.034
	Model 2	State anxiety (STAI)	0.35	0.08	2.65	0.011
		Sensorial Pain (MPQ)	−0.29		−2.19	0.033

Note: d2 = d2 Attention test; D-KEFS = Delis–Kaplan Executive Function Test Battery Trail Making Test; ROCF = Rey–Osterrieth Complex Figure Test; 5DT = Five Digit Test; TAVEC = Test de.

## Data Availability

The authors claim that this manuscript describes an original research work which has not been preregistered. The data presented in this study are available on request from authors. The data are not publicly available due to compliance with privacy laws.
